# Hepatic Candidiasis in a Non-neutropenic Patient

**DOI:** 10.7759/cureus.101055

**Published:** 2026-01-07

**Authors:** Maria Carolina Carvalho, Matilde Coimbra, Micaela Caixeiro, Ricardo Paquete Oliveira, José Delgado Alves

**Affiliations:** 1 Department of Internal Medicine IV, Hospital Prof. Dr. Fernando Fonseca, Amadora, PRT; 2 Department of Infectious Diseases, Hospital Prof. Dr. Fernando Fonseca, Amadora, PRT; 3 Immune-Mediated Systemic Diseases Unit, Hospital Prof. Dr. Fernando Fonseca, Amadora, PRT

**Keywords:** candida, chronic disseminated candidiasis, corticosteroids, hepatic candidiasis, invasive candidiasis

## Abstract

Hepatic candidiasis is an uncommon but serious manifestation of invasive candidiasis (IC), most often associated with prolonged neutropenia in patients with hematological malignancies or in those who have undergone abdominal surgery. Diagnosis is challenging because of the low sensitivity of cultures, the limitations of serologic assays, and the frequent impracticality of tissue biopsy. Imaging, particularly CT and [18F]fluoro-2-deoxy-D-glucose positron emission tomography (18FDG-PET), plays a central role in identifying characteristic hepatosplenic lesions and in monitoring response to therapy. We present a case of hepatic candidiasis in a non-neutropenic patient presenting with persistent fever and multiple risk factors for IC, highlighting the diagnostic complexity and the importance of maintaining clinical suspicion beyond the classical neutropenic setting. Management requires prompt initiation of appropriate antifungal therapy, thorough evaluation for secondary foci of infection, and prolonged treatment until complete radiologic resolution is achieved to prevent relapse. Current recommendations for the management of deep-seated candidiasis remain largely based on retrospective data and expert opinion. Further research is needed to optimize diagnostic strategies, define the role of molecular diagnostic techniques, and identify patients with underlying genetic susceptibilities that may predispose them to invasive disease.

## Introduction

Invasive candidiasis (IC) is the leading cause of healthcare-associated fungal infections in high-income countries and represents a significant source of morbidity and mortality among hospitalized patients. It encompasses both candidemia (bloodstream infection) and, less commonly, disseminated candidiasis with organ involvement, usually affecting the abdomen, bone, heart, or eye, which may occur simultaneously or independently. There are at least 15 Candida species capable of causing human disease, but more than 90% of invasive infections are attributed to six predominant pathogens: C. albicans, C. glabrata, C. tropicalis, C. parapsilosis, C. krusei, and, in some regions, C. auris. Although C. albicans remains the most common species, over half of IC cases are now caused by non-albicans species [1,2]. The principal risk factors associated with IC include ICU admission, prolonged neutropenia, particularly in patients with hematological malignancies, exposure to broad-spectrum antibiotics, and abdominal surgery. These factors explain why nearly 60% of cases occur in ICUs and approximately 13% in cancer and transplant units [1-3].

Although candidemia accounts for most IC cases, disseminated candidiasis can occur with blood culture-negative disease and presents with a broad spectrum of clinical manifestations depending on the site of focal involvement. Diagnostic complexity is further increased in neutropenic patients, in whom signs of focal infection, such as hepatosplenic abscesses or abnormalities on funduscopic examination, may not be evident until neutrophil recovery, necessitating repeated evaluations [1]. As the hospital-acquired infection with the highest mortality rate, with a 30-day post-diagnosis mortality of 40-55%, a high index of suspicion is essential to ensure prompt initiation of therapy and improve patient outcomes [3]. We present a rare case of hepatic candidiasis in a non-neutropenic patient.

This report was previously presented as a poster at the Portuguese National Congress of Internal Medicine, in Porto, Portugal, in May 2023.

## Case presentation

**Figure 1:**
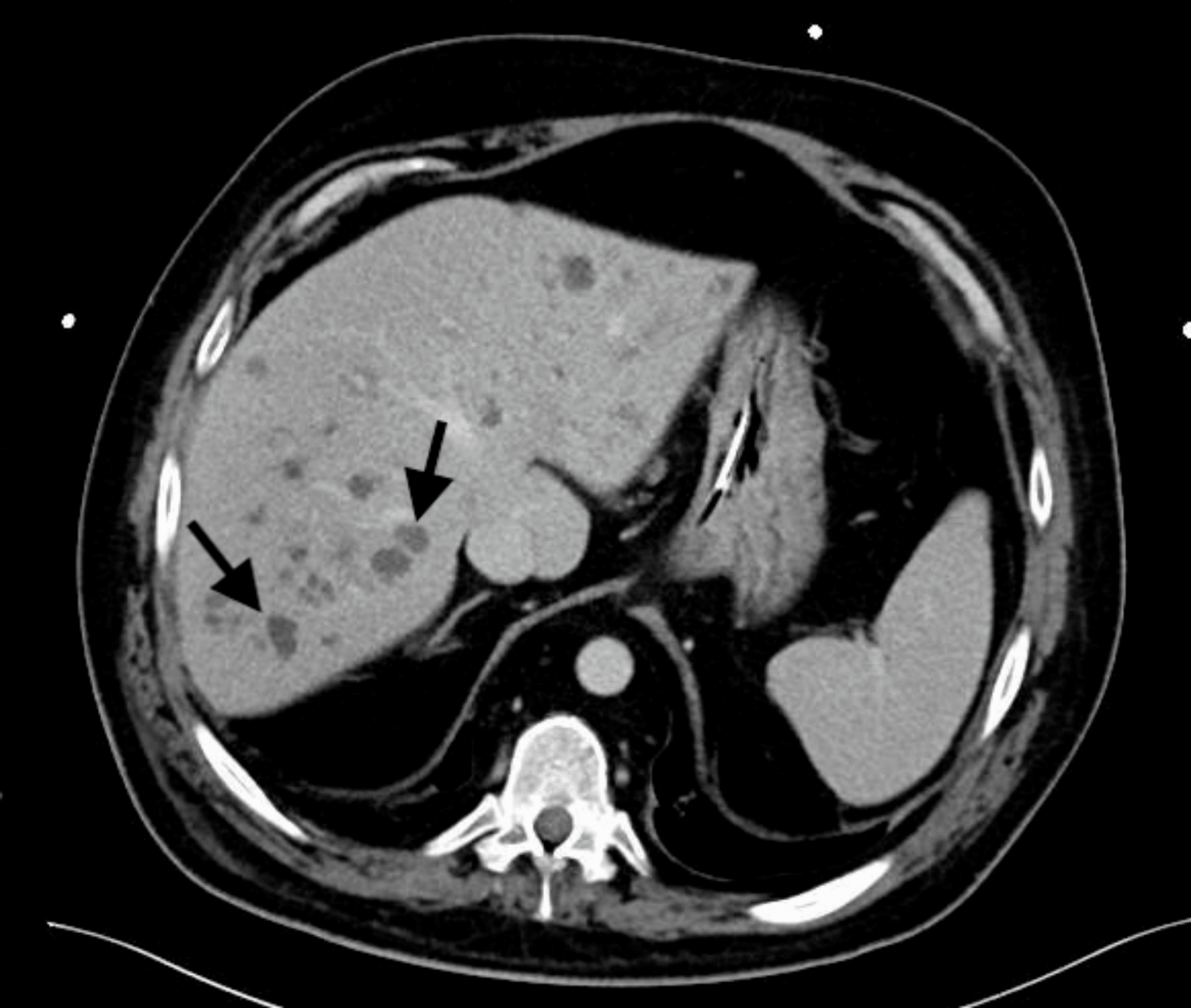
Abdominal CT scan showing multiple hepatic hypodense lesions (arrows) CT: computed tomography

A 48-year-old male with a medical history of corticosteroid-dependent asthma (10 mg/day of prednisolone) and bronchiectasis was admitted to the ICU for status asthmaticus. During his ICU stay, he experienced multiple infectious complications caused by multidrug-resistant bacteria, as well as candidemia, with isolation of C. albicans in one set of blood cultures. He was treated with caspofungin for 21 days, which was discontinued 10 days after negative blood cultures. One month after discontinuation, and following his transfer to an Internal Medicine ward, he developed cholestasis, elevated inflammatory markers, and fever, with persistently negative cultures, including those from the central venous catheter, which was replaced. An abdominal CT scan revealed multiple nodular hypodense hepatic lesions that were not present at the time of admission (Figure 1).

Although the patient never developed neutropenia, given multiple other risk factors - including previous candidemia, immunosuppression from long-term corticosteroid therapy, exposure to broad-spectrum antibiotics, ICU admission, and the presence of a central venous catheter - the presumptive diagnosis of hepatic candidiasis was made, and antifungal therapy was reinitiated with echinocandins. Ophthalmologic, cardiac, and vascular foci were excluded, and repeat blood cultures remained negative. A liver biopsy revealed neutrophilic microabscesses, as well as areas of necrosis and granuloma formation.

**Figure 2:**
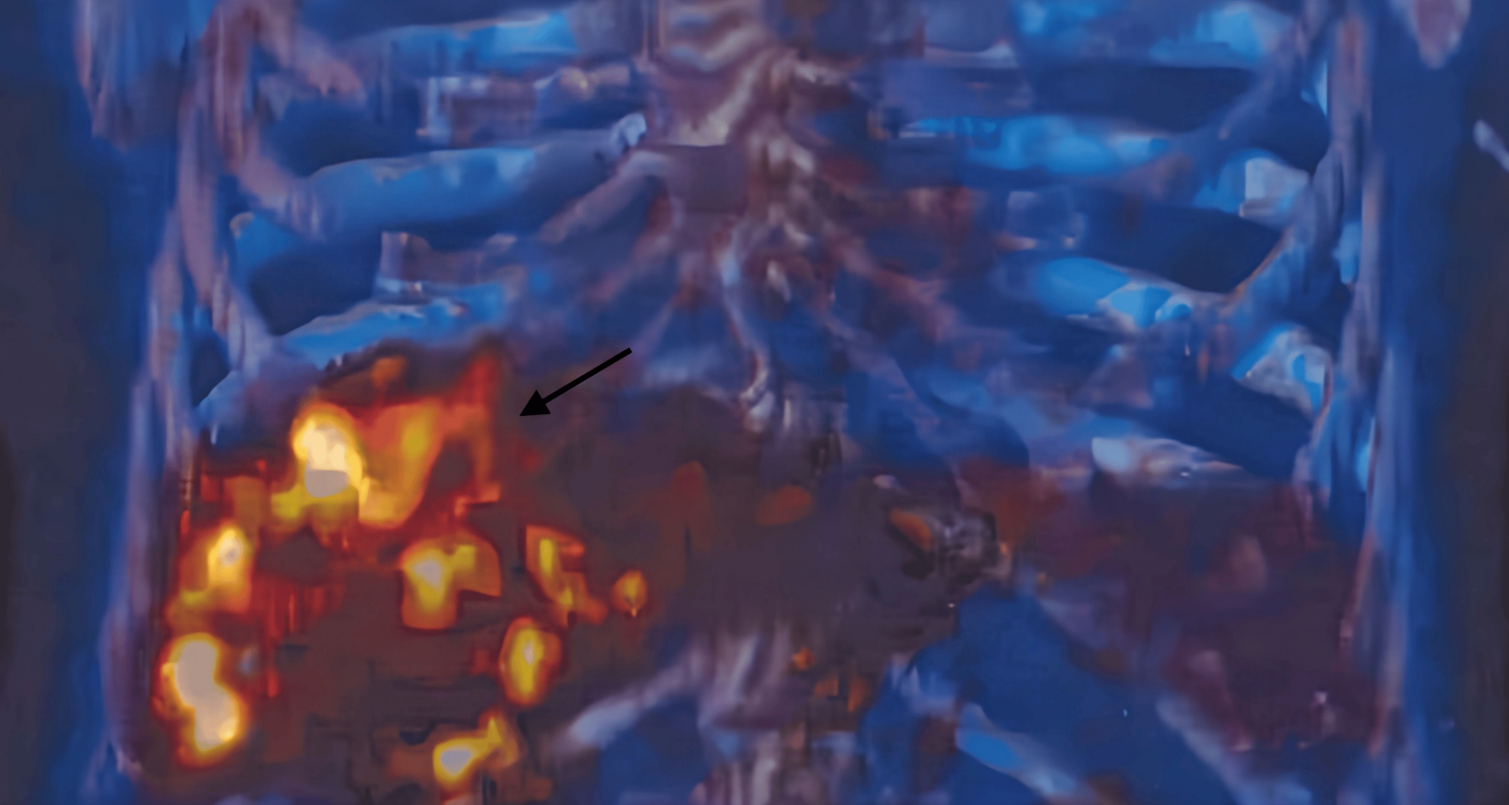
PET scan of the abdomen showing hypermetabolic hepatic lesions (arrow) PET: positron emission tomography

Although fungal cultures were negative and no organisms were observed, a panfungal polymerase chain reaction (PCR) test detected C. albicans. Considering the persistently negative cultures and clinical improvement-including persistent apyrexia and resolution of cholestasis-therapy was switched from caspofungin to oral fluconazole before discharge. Despite clinical and imaging improvement over the following months, follow-up CT scans showed persistent lesions, although markedly reduced in size. After six months of therapy, a fluorine-[18F]fluoro-2-deoxy-D-glucose positron emission tomography (18FDG-PET) scan was performed, demonstrating hypermetabolic hepatic lesions and confirming ongoing disease activity (Figure 2).

Currently, after nearly four years of therapy, the patient continues oral antifungal treatment due to persistent hepatic lesions and residual cholestasis.

## Discussion

Chronic disseminated candidiasis, also known as hepatosplenic candidiasis (HSC), refers to cases of invasive candidiasis with involvement of the liver and/or spleen. Because Candida species commonly colonize the skin and gastrointestinal tract, disruptions of the mucosal microbiota or host immunosuppression can allow the organism to shift from a commensal to a pathogenic state. Several well-established risk factors for this transition are summarized in Table 1 [1,2].

**Table 1:** Risk factors for invasive candidiasis

Risk factors for invasive candidiasis
Immunosuppression (particularly prolonged neutropenia and active malignancies)
Hospitalization in intensive care units
Central vascular catheters
Parental nutrition
Mechanical ventilation
Hemodialysis
Invasive abdominal procedures (especially abdominal surgery with subsequent anastomotic leakage)
Broad-spectrum antibiotic therapy
Solid organ transplantation
Hematopoietic stem cell transplantation
Acute necrotizing pancreatitis
Colonization by Candida spp.

Although candidemia is relatively common in these settings, deep-seated candidiasis, with or without concurrent candidemia, is far less frequent [1,3]. HSC occurs predominantly in immunosuppressed patients, particularly those with severe and prolonged neutropenia. For this reason, it is most often observed in patients receiving chemotherapy for hematologic malignancies, and with the introduction of antifungal prophylaxis in this population, its incidence has declined. HSC is even less common in non-neutropenic individuals, and when it occurs, it is usually in patients with a history of abdominal surgery, recurrent gastrointestinal perforations, or anastomotic leaks [3-5].

Although our patient was considered immunosuppressed due to long-term corticosteroid therapy, only doses equivalent to at least 20 mg of prednisolone per day are typically regarded as significantly increasing the risk of IC, whereas the patient’s maintenance dose was 10 mg/day [6,7]. However, this risk may have been amplified by additional factors, including ICU hospitalization, the presence of invasive vascular devices, and exposure to broad-spectrum antibiotics, which likely contributed to the development of IC. Notably, despite sharing similar risk factors, most patients with prolonged ICU stays do not develop IC. This observation suggests that individual genetic variations may increase susceptibility or provide protection against severe infections, and recent studies have indicated that genetic polymorphisms may influence individual risk [1,3,8].

As demonstrated in the present case, the diagnosis of IC is often challenging. Although positive cultures remain the gold standard, blood culture sensitivity in candidemia ranges from 63 to 83% and decreases substantially in cases of deep-seated candidiasis, with reported sensitivities between 21% and 71%. Sensitivity also varies depending on the Candida species involved and prior antifungal exposure [1,3,9]. In HSC, a definitive diagnosis still requires histological or microbiological confirmation from a tissue biopsy demonstrating fungal infection. In practice, however, the risks associated with liver or spleen biopsy, particularly in patients with thrombocytopenia, who make up a large proportion of at-risk individuals, render this approach impractical in many cases. Moreover, tissue cultures are positive in approximately half of the patients. Liver biopsy specimens typically show one of three histologic patterns: microabscesses with intense inflammation (as in the present case, often detectable only after neutrophil recovery in neutropenic patients), necrosis with minimal inflammation, and granulomas [4].

These diagnostic challenges have driven efforts to improve non-cultural methods for diagnosing IC. The introduction of molecular techniques, such as PCR-based assays, has facilitated the detection of Candida DNA in tissue samples and should be considered when available. However, due to the heterogeneity of available assays, none have been fully validated for the diagnosis of IC [10,11]. Other diagnostic approaches include serum assays for Candida antigens and anti-Candida antibodies, but these generally have low sensitivity in immunosuppressed patients and cannot distinguish acute infection from prior exposure. Tests for mannan antigen and antimannan IgG, as well as β-D-glucan assays, which detect fungal cell wall components, have been validated as useful tools, but their performance varies widely depending on the specific assay used in each laboratory, and they cannot differentiate among Candida species. Importantly, most of these tests were evaluated in the context of candidemia, and their sensitivity is significantly lower in deep-seated infections, particularly in HSC [10].

Given these diagnostic difficulties, a definitive diagnosis is uncommon, and most cases of IC are classified as probable based on clinical findings, imaging, and direct or indirect evidence of fungal infection. Typical CT findings in HSC include multiple hypodense nodular lesions, as observed in this case. 18FDG-PET scans are more sensitive than CT for detecting hepatosplenic lesions, and PET uptake often resolves earlier than CT abnormalities. Studies suggest that monitoring disease activity with 18FDG-PET approximately three months after starting therapy can help guide decisions about the appropriate timing for discontinuing treatment [4,12]. These challenges are well illustrated by the present case. Despite multiple risk factors, hepatic lesions, and histopathological features consistent with hepatic candidiasis, both blood and tissue cultures were negative, creating uncertainty regarding the diagnosis. Nevertheless, considering the history of prior candidemia along with these additional findings, the diagnosis of HSC was maintained, and antifungal therapy was initiated without delay. This approach allowed time to pursue alternative methods of diagnostic confirmation, which was ultimately achieved through panfungal PCR testing, highlighting its diagnostic value.

Regarding treatment, although guidelines exist for invasive candidiasis, most studies have focused on candidemia, and recommendations for managing HSC are based largely on retrospective case series and anecdotal experience. According to the most recent guidelines (2016), management is divided into two main groups: neutropenic and non-neutropenic patients. In non-neutropenic patients, first-line therapy is an echinocandin (typically caspofungin, although any echinocandin may be used), and fluconazole is considered a second-line option, particularly for clinically stable patients. For those started on an echinocandin, step-down therapy to fluconazole is recommended once the patient is clinically stable, the isolate is fluconazole-susceptible, and blood cultures have cleared [13].

Although empiric antifungal therapy is not routinely recommended due to concerns about antifungal resistance, initiating treatment before diagnostic confirmation may be considered in patients with multiple risk factors for IC and persistent fever without an identifiable bacterial source. Early empiric therapy has been associated with improved clinical outcomes, although it has not been shown to reduce overall mortality [14]. Exclusion of other possible sites of infection should also be considered. All non-neutropenic patients with candidemia should undergo ophthalmologic examination within the first week after diagnosis. The estimated incidence of ocular involvement is around 35%, although some authors report rates as low as 5% and argue that screening should be limited to symptomatic or non-communicative patients. Nonetheless, given the potentially severe consequences of Candida endophthalmitis, current guidelines continue to strongly recommend routine screening.

Evaluation for other secondary sites of infection is not routinely recommended unless clinically indicated, except for Candida endocarditis. Although echocardiography is not mandatory for all patients, clinicians should maintain a low threshold for investigation; persistent candidemia, even in the absence of new murmurs or fever, warrants assessment to exclude cardiac involvement. Additional recommendations include obtaining follow-up blood cultures daily or every other day until clearance and removing intravascular catheters in all non-neutropenic patients with candidemia, even when the catheter is not suspected to be the source [11,13]. In patients with persistent fever despite appropriate antifungal therapy, short courses of nonsteroidal anti-inflammatory drugs or corticosteroids may be considered. For HSC specifically, therapy should be continued until radiological resolution of lesions, as premature discontinuation may result in relapse. Although evidence regarding the optimal duration is limited, consensus supports prolonging treatment until imaging findings have resolved - typically around six months, but as demonstrated in this case, sometimes for several years [13].

## Conclusions

Hepatosplenic candidiasis remains a rare but clinically important manifestation of invasive candidiasis, presenting significant challenges in diagnosis and management due to its nonspecific clinical presentation, limited sensitivity of conventional diagnostic tools, and reliance on imaging and indirect microbiological evidence. This report emphasizes the importance of maintaining a high index of suspicion in at-risk patients, including those without classic neutropenia, and highlights the need for a multimodal diagnostic approach that integrates clinical, radiological, and laboratory findings. Early initiation of appropriate antifungal therapy, careful monitoring for complications, and prolonged treatment until radiologic resolution are essential to prevent relapse. Because current recommendations for HSC are largely based on retrospective data and expert opinion, further studies are urgently needed to refine diagnostic strategies, clarify the role of emerging molecular and imaging techniques, and identify patients with underlying genetic susceptibilities that may increase the risk of invasive disease. Such advances will be crucial for improving early recognition, individualizing management, and ultimately reducing morbidity associated with this uncommon but serious infection.
